# A single group follow-up study of non-surgical patients seen by physiotherapists working in expanded roles in orthopaedic departments: recall of recommendations, change in exercise and self-efficacy

**DOI:** 10.1186/1756-0500-5-669

**Published:** 2012-12-04

**Authors:** Crystal MacKay, Aileen M Davis, Nizar N Mahomed, Elizabeth M Badley

**Affiliations:** 1 Division of Health Care and Outcomes Research and Arthritis Community Research and Evaluation Unit, Toronto Western Research Institute, Toronto, ON, Canada; 2 Department of Physical Therapy, University of Toronto, Toronto, ON, Canada; 3 Department of Rehabilitation Science, University of Toronto, Toronto, ON, Canada; 4 Institute of Health Policy, Management and Evaluation, University of Toronto, Toronto, ON, Canada; 5 Arthritis Program, Toronto Western Hospital, University Health Network, Toronto, ON, Canada; 6 Department of Surgery, University of Toronto, Toronto, ON, Canada; 7 Dalla Lana School of Public Health, University of Toronto, Toronto, ON, Canada

**Keywords:** Arthritis, Physiotherapists, Advanced practice, Non-surgical, Self-management behaviours, Orthopaedics

## Abstract

**Background:**

Specially trained physiotherapists (advanced practice physiotherapists (APP)) are working in orthopaedic clinics to improve access to orthopaedic services and support chronic disease management. Little attention has been paid to the impact APPs may have on non-surgical patients. In non-surgical patients with hip or knee arthritis consulting an APP in an orthopaedic clinic, the objectives were to: 1) describe patients’ recall of APP recommendations, use of self-management strategies, and barriers to management six weeks following consultation; and, 2) compare exercise behaviour and self-efficacy at baseline and six weeks.

**Findings:**

This was a single group pre-and post-intervention study of patients who saw an APP when consulting the orthopaedic departments of two hospitals. At baseline and six weeks participants completed the adapted Stanford Exercise Behaviour Scale (response options: none, < 60 minutes/week, 1–3 hours/week or > 3 hours/week), and the Chronic Disease Self-efficacy Scale (range 1–10; higher scores indicate higher self-efficacy). At follow-up participants completed questions on recall of APP recommendations, use of self-management strategies and barriers to management. Seventy three non-surgical patients with hip or knee arthritis participated, a response rate of 89% at follow-up. Seventy one percent of patients reported that the APP recommended exercise, of whom 83% reported exercising to manage their arthritis since the visit. Almost 50% reported an increase in time spent stretching; over 40% reported an increase in time spent walking or doing strengthening exercises at follow-up. Common barriers to arthritis management were time, cost and other health problems. Mean chronic disease self-efficacy scores significantly improved from 6.3 to 7.2 (p < 0.001). The mean difference was 0.95 (95% CI 0.43, 1.62); the effect size was 0.51.

**Conclusions:**

This pilot study of an APP intervention for non-surgical patients referred for orthopaedic consultation showed promising results, particularly for enhancing use of conservative management strategies such as exercise.

## Findings

### Background

The increasing prevalence of arthritis [1] and concomitant concerns particularly about access to total joint replacement surgery (TJR), including long wait times, geographic variations in access, and barriers to services, have provided impetus for the development of alternative ways of delivering orthopaedic services [2-9]. Increasingly, health professionals, such as specially trained physiotherapists, are working in expanded roles in orthopaedic settings. Research to date on the impact of specially trained physiotherapists in orthopaedic clinics has focused largely on the effectiveness of physiotherapists triaging patients to surgery including examination of wait times for surgery, surgical conversion rates and physiotherapist decision-making in comparison to the surgeon’s clinical decisions [10-16]. However, little attention has been paid to the relatively high proportion of patients referred to surgeons who require non-surgical musculoskeletal management [17-20] and the potential for specially trained physiotherapists to affect outcomes in this group by providing education and advice on conservative management. Exercise has been shown to reduce pain and improve function in people with arthritis [21-24]. Increased self-efficacy has been shown to be one mechanism for improving health status [25,26]. It is unclear whether consultation with a specially trained physiotherapist results in any changes to self-management behaviours, such as exercise or self-efficacy.

Relating to patients with hip or knee arthritis who visited an orthopaedic clinic for consideration of TJR, the objectives of the study were to: 1) describe patients’ recall of APP recommendations, use of self-management strategies such as exercise, and barriers to management six weeks following the consultation; and, 2) compare exercise behaviour and self-efficacy in patients at baseline to six weeks after their consultation with the APP.

## Methods

This was a single group pre-and post-intervention pilot study in the orthopaedic departments of two tertiary care urban teaching hospitals in Toronto, Ontario, Canada.

### Participants

The study sample consisted of patients with hip or knee arthritis referred for orthopaedic surgeon consultation for consideration of TJR who were determined to be non-surgical on consultation in the orthopaedic clinic. Inclusion criteria were a diagnosis of arthritis, non-surgical status (i.e. not requiring any type of orthopaedic surgery) and fluency in oral and written English. Exclusion criteria were referral from other orthopaedic surgeons (these patients were seen directly by the orthopaedic surgeon); follow-up patients who had been seen previously in the orthopaedic clinic; patients referred for consideration of a revision TJR; and patients with musculoskeletal injuries including acute musculoskeletal injury, recurrent trauma with no mention of arthritis, or evidence of meniscal/ligamentous injury in patients under age 45.

### Procedures

A research coordinator screened patients for eligibility for the study. Written informed consent was obtained from all study participants. Consenting participants completed questionnaires in the orthopaedic clinic during their visit while waiting to be seen. In the clinic, patients were assessed by one of four specially trained physiotherapists (APPs). In addition to on-the-job training, all APPs had completed or were in the process of undertaking a clinical-education training program in advanced musculoskeletal/arthritis care. The clinical encounter with the APP included a detailed history, musculoskeletal examination, and review of diagnostic imaging such as radiographs and magnetic resonance imaging. Patients assessed as likely to need surgery were sent on for consultation with an orthopaedic surgeon [14]. For patients with arthritis who were deemed non-surgical APPs provided education on conservative management strategies. Although the intervention was not standardized, the types of conservative management strategies provided to non-surgical patients included, as required, education regarding their type of arthritis, activity modifications, use of assistive devices such as braces, use of gait aids as a means of joint protection, and weight management. These were supplemented by educational hand-outs and referral to web-based materials as needed. Instructions related to specific types of exercise (e.g. strengthening exercises, stretches and aerobic exercise) were also provided. Patients who required ongoing support for disease management were referred to community services, such as outpatient physiotherapy, occupational therapy, specialized arthritis programs, aquatic programs or arthritis self-management programs.

Patients were followed at six weeks after their visit using a structured telephone interview. Prior to the six week telephone follow-up, participants were mailed a reminder letter and standardized interview questionnaires that allowed them to follow the interview questions. Qualitative data from the telephone interviews are not reported in this paper. All procedures were approved by the University Health Network Research Ethics Board and Mount Sinai Research Ethics Board.

### Measures

Baseline questionnaires completed in clinic were as follows: a) A measure of symptoms and functional status (the Knee Injury and Osteoarthritis Outcome Score (KOOS) or Hip Dysfunction and Osteoarthritis Outcome Score (HOOS)) depending on the primary joint affected. The KOOS and HOOS have been shown to be valid, reliable and responsive in people with hip and knee OA [27,28]. The KOOS/HOOS each have five subscales with 5-point Likert scales for response options. Summed subscale scores were transformed to a percentage with higher scores indicating better function [29]. b) The Self-Efficacy for Managing Chronic Disease 6-Item Scale, a six item scale where respondents rate their confidence on a 1–10 scale with 1 being not at all confident and 10 being totally confident. The score for the scale is the mean of the six items. c) An adaptation of the Stanford Exercise Behaviour Scale which asks participants how much total time for the entire week they spent on different types of exercise. The response options were modified to include 4 options: none, < 60 minutes/week, 1–3 hours/week or > 3 hours/week. d) Questions on demographics (i.e. age, sex, presence of comorbidities, education, employment, and living arrangement).

At six week follow-up, participants had a structured telephone administered interview that addressed recall of specific APP recommendations, what participants were currently doing to manage their arthritis and barriers to arthritis management. Participants also completed the adapted Stanford Exercise Behaviour Scale and Self-Efficacy for Managing Chronic Disease 6-Item Scale in order to compare to baseline measures.

### Statistical analysis

Descriptive analyses were conducted to describe the study sample and questionnaire responses. Paired t-tests were used to examine self-efficacy at baseline and follow-up and the effect size was calculated to examine the magnitude of the effect. The effect size was calculated as the mean difference over the pooled standard deviation. The proportion of patients who reported an increase (increase by at least one category of time), decrease (decrease by at least one category of time) or no change in exercise was calculated using the Exercise Behaviour Scale responses at the two time points. Due to small numbers reporting time in these activities, three variables were combined into ‘other aerobic exercise’ in the analysis (bike, swim or other aerobic exercise). The total time spent in these activities was used to calculate change in exercise over time.

## Results

Between September 2007 and December 2008, 301 participants were approached to participate in the study. Of these, 199 adults with hip or knee arthritis who attended the clinic for consideration of surgery met preliminary inclusion criteria and gave consent to participate in the study. Following assessment, eighty seven individuals (43.7%) were deemed non-surgical and included in this study. At follow-up, 73 participants responded to the telephone follow-up for a response rate of 83.9%. Of the non-respondents, 11 were lost to follow-up (i.e. we were unable to make contact with the participants by telephone) and three individuals refused further participation. There were some missing responses at baseline related to self-efficacy and exercise behaviour and demographic characteristics likely related to feasibility of completing the full questionnaire while waiting to be seen. At least 85% of data were available for each question. Only available data were used in the analysis.

Of the 73 patients with baseline and follow-up questionnaires, 60 patients had been referred for orthopaedic consultation for knee problems, 12 for hip problems and one person was referred for both hip and knee problems. Sixty seven percent were female and the mean age was 58.5 years (age range 19–82). Over 70% had at least one comorbidity with low back pain being the most common (n = 28). Other chronic conditions reported were: high blood pressure (n = 11), depression (n = 10), asthma (n = 7), cancer (n = 5) and anemia (n = 5). Table 1 shows the patient characteristics. The lowest scores on the HOOS and KOOS were in the Sport and Recreation and Hip or Knee Related Quality of Life subscales indicating greater difficulty in these domains (Table 2).

**Table 1:** Patient characteristics

***Demographics***	**N**	
**Age:** mean (range)	73	58.5 (19–82)
**Female**	73	49 (67.1%)
**Comorbidity**	65	47 (72.3%)
**Education**	66	
Less than high school/high school		23 (34.8%)
Trades certificate/Diploma/College		18 (27.3%)
University		25 (37.9%)
**Living arrangement**	68	
Lives with other person		54 (80.0%)
**Employment**	68	
Working		43 (63.2%)
Not working (Unemployed, disabled or retired due to ill health, student)		8 (11.8%)
Homemaker/retired		17 (25.0%)

**Table 2:** Baseline patient function

***Patient function***	***N***	***Mean (SD)***
**KOOS Subscale**	N = 61*^+^	
Pain		50.6 (19.3)
Other Symptoms		53.5 (21.4)
Activities of Daily Living		56.9 (19.9)
Sport and Recreation		33.1 (25.0)
Knee Related Quality of Life		31.2 (19.9)
**HOOS Subscale**	N = 13*^++^	
Pain		62.8 (22.1)
Other Symptoms		59.0 (20.4)
Activities of Daily Living		67.4 (22.5)
Sports and Recreation		58.3 (29.0)
Hip Related Quality of Life		49.8 (21.2)

*one participant was referred for hip and knee complaints and completed both the HOOS and KOOS.

+range of valid numbers completing subscales was 57–61.

++range of valid numbers completing subscales was 12–13.

At six weeks, 89% of participants reported that the APP made at least one recommendation for their arthritis management. The majority of participants (71%) reported that the APP had specifically recommended exercise and 64% of participants reported that the APP reviewed information on arthritis with them (Table 3). Fewer participants reported that the APPs had recommended they contact The Arthritis Society (32%) (a not-for-profit organization devoted to funding and promoting arthritis research, programs and patient care), lose or manage their weight (21%), use assistive devices (18%) or take a course or class (12%). Eighty three percent of people who recalled an APP recommendation for exercise reported using the strategy for their arthritis management at six week follow-up. Overall, the most common strategies participants reported using to help manage their arthritis at six weeks were: exercise (76%), weight loss or weight management (37%), and reviewing information on arthritis (37%) (Table 3). Participants also reported barriers to managing their arthritis; the most common barriers identified by participants were not enough time (37%), cost of services or equipment (25%), and other health problems (25%) (Table 4).

**Table 3:** Recall and uptake of recommendations (n = 73)

	**Number (%) who reported APP recommended strategy**	**Of those who recalled recommendation, number (%) who used strategy**	**Overall number (%) who reported using strategies to manage arthritis at 6 weeks**
Exercise	52 (71)	43 (83)	56 (76)
Review information on arthritis	47 (64)	20 (43)	27 (37)
Contact The Arthritis Society*	23 (32)	5 (22)	6 (8)
Weight loss/management	15 (21)	11 (73)	27 (37)
Use assistive devices	13 (18)	7 (54)	17 (24)
Take a course or class	9 (12)	1 (11)	2 (3)

*A not-for-profit organization devoted to funding and promoting arthritis research, programs and patient care.

**Table 4:** Barriers to arthritis management (n = 73)

**Barriers**	**Number/percent**
Not enough time	27 (37.0)
Costs of services or equipment	18 (24.7)
Other health problems	18 (24.7)
Lack of availability of services	9 (12.3)
Difficulty remembering what to do	8 (11.0)
Transportation	7 (9.6)

**Figure 1:**
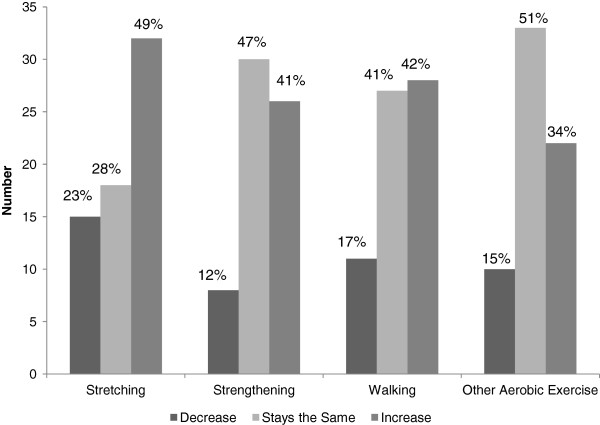
Pre-Post Change in Exercise (Time Spent Exercising for the Past Week) (N = 66*). About 50% of patients increased time spent stretching, and over 40% increased the time spent in walking or doing strengthening exercises. Only a minority of patients decreased the time spent in stretching, strengthening exercises or walking. *range in valid numbers reporting type of exercise was 64–66.

Self-reported time spent in different types of exercise at baseline was compared to follow-up measures. About 50% of patients had increased time spent stretching, while 28% stayed the same and 23% decreased. Over 40% had increased the time spent in walking while 41% stayed the same and 17% decreased. Over 40% increased time spent doing strengthening exercises while 47% stayed the same and 12% decreased. Thirty four percent of patients increased the time spent in other aerobic exercises, 51% remained the same and 15% decreased (Figure 1).

The mean overall self-efficacy score at baseline was 6.3; at six weeks, there was an increase in the overall mean score of one point to 7.2, a statistically significant improvement (p < 0.0005). The mean difference was 0.95 (95% confidence interval 0.43, 1.62). The effect size for self-efficacy was 0.51, which indicates a medium effect. All six items in the self-efficacy scale showed improvement, with significant increases in self-efficacy for managing fatigue, pain, emotional distress and other health problems.

## Discussion

This pre-post-intervention study followed non-surgical arthritis patients for six weeks following a pilot intervention in which an APP provided assessment and consultation within orthopaedic clinics. One of the most notable findings was the increase in exercise reported by some patients at follow-up, where over 40% reported an increase in time spent in exercise. We also found significant improvement in self-efficacy on follow-up. The intervention was a one visit model, without further follow-up. We acknowledge that this as well as the absence of a control group or retrospective audit is a key limitation which means we cannot firmly interpret these results as an effect of the APP recommendations. Nevertheless, a high proportion of patients recalled that the APP had recommended exercise for their arthritis and were engaging in exercise behaviours at six weeks. In other research, a recommendation from a health professional to exercise has been shown to be the strongest predictor of recent exercise/physical activity in adults with arthritis [30]. Improvements in exercise are important as aerobic and strengthening exercise has been shown to improve pain, physical function and psychological outcomes in people with arthritis [21,22,31]. With the increasing emphasis on chronic disease management, these findings are a first step in suggesting potential alternative ways of providing support for management to non-surgical patients with arthritis.

Usual care in orthopaedic clinics in Canada commonly consists of an orthopaedic surgeon working independently in clinics seeing patients referred from primary care physicians. Prior research demonstrates that orthopaedic surgeons are less likely to provide education and exercise in the clinic than an APP [14]. McHugh also found that patients seeing an orthopaedic specialist were not provided with information on exercise in almost 60% of cases or information on pain management in 65% of cases [32]. In this setting, APPs assess patients and provide advice on conservative management; all surgical patients are seen by an orthopaedic surgeon. This study provides preliminary insights into the impact of this role in orthopaedic care.

While some participants reported increasing their exercise time at 6 weeks, some were unchanged in their exercise behavior. This highlights the need for further research to address how best to identify individuals less likely to change their behaviour. Furthermore, it suggests further improvements can be made to increase the adoption of positive self-management behaviours. Health literacy has been identified as a significant problem [33] with some evidence it is linked to limited self-management skills and poor health outcomes [34-37]. Briggs et al. examined the health literacy among individuals with chronic low back pain and without low back pain using the Health Literacy Measurement Scale [38]. They found that individuals with low back pain had a significantly lower score in the domain ‘patient attitudes towards their health’ (i.e. ability to attend to their health needs and willingness to change lifestyle or adapt behaviour) [38]. The authors suggest that self-management support initiatives may benefit individuals with low back pain and that care providers should consider exploring individuals’ attitudes towards their health and barriers to using self-management strategies. In a recent systematic review there is some evidence that interventions designed to mitigate the effects of low health literacy, and intensive mixed-strategy interventions focusing on self-management, improve health care and disease outcomes [39]. Understanding and addressing health literacy could be considered as one strategy for further improving use of positive self-management strategies such as exercise.

Patients most commonly reported APP recommendations for exercise while other strategies, such as weight loss or use of assistive devices, were recalled less frequently. This may not be surprising as exercise is a key best practice for arthritis management [40] and physiotherapists are considered expert in exercise prescription and management of pathology related to exercise [41]. It is unclear to what extent other strategies, such as gait aids were relevant given the disease stage and symptoms of this non-surgical group. Data on body mass index were not collected and it is also unclear what percentage of the sample may have benefited from consultation on weight management.

In our study, self-efficacy for managing a chronic disease also improved significantly and the effect size showed a medium effect. While we are unaware of research that would suggest what a meaningful difference in self-efficacy would be (e.g. MCID), self-efficacy has been measured in other research on non-pharmacologic interventions for arthritis and other chronic diseases, such as exercise and self-management programs [21,22,26,42-46]. The effect size in our research was similar to other research using this measure. For example, Griffiths examined the Chronic Disease Self-Management Program and found an improvement in self-efficacy (effect size 0.67) [47]. Gitlin found a 0.6 mean change in self-efficacy following a Chronic Disease Self-Management Program which was statistically significant and considered to be an improvement [48]. While we don’t know if the increase in participants’ confidence to manage their condition will result in longer term change in outcomes, self-efficacy has been shown to relate to adaptive pain behaviours in arthritis [49].

While exercise behaviour and self-efficacy showed some improvement in our sample, participants also reported barriers to managing their arthritis as recommended. The most common barriers were time, cost of services/equipment and other health problems. These barriers may need to be addressed to optimize patient management of their arthritis. Previous studies on barriers to exercise and/or physical activity in people with arthritis and in the general population support these findings [50-52]. While participants indicated care for other health conditions can be barriers to their arthritis management, it is possible, in turn, that lack of adequate arthritis management, may affect outcomes of their other conditions (e.g. due to difficulty engaging in physical activity), illustrating the importance of a holistic approach to the management of the health of individuals.

There is little prior research in the literature on the impact of specially trained physiotherapists working in orthopaedic care on patients. One of the few studies examining patient outcomes was a study conducted in the United Kingdom which examined pain, function and handicap in a randomized controlled trial of both surgical and non-surgical patients; the researchers found no significant differences between groups of orthopaedic patients randomized to see a specially trained physiotherapist or post-fellowship junior staff and clinical assistant orthopaedic surgeons [53]. There is a dearth of literature specific to non-surgical patients referred for surgical consultation for TJR.

The findings of our study also raise broader questions about the role of APPs in health care delivery. While this pilot study focused on an APP intervention for non-surgical patients with arthritis in orthopaedic clinics in a tertiary care environment, further research is required to understand the potential impact of APP support for arthritis management in other clinical settings, such as primary care where the vast majority of patients are seen [54]. Primary care may provide an opportunity for earlier intervention and secondary prevention of arthritis pain and disability and potentially delay need for surgical consultation in addition to triaging appropriate patients to orthopaedic surgeons and other specialists.

While self-management support has often focused on group-based self-management programs such as those developed by Lorig [26,44,55-58], there is an increasing emphasis on health care providers supporting self-management in clinical practice . While our results need to be interpreted with caution, the improvements in exercise behaviours and self-efficacy in our study, albeit short-term, suggest there may be potential that minimal interventions by health care providers may impact positively on patient self-management and confidence. Further research is required to confirm these findings. We also need to understand which types of patients may benefit from more intensive interventions versus those who may have improved outcomes from shorter interventions such as the intervention described in this study.

Advanced roles for physiotherapists are a relatively recent development in Canada and continue to evolve. Health care is a provincial responsibility as are the regulatory frameworks governing health professionals. Ontario, Canada’s largest province, has embraced expansion of roles of rehabilitation professionals. The term APP is not a formal title provided by the regulatory body but is one chosen by most hospitals in Ontario with physiotherapists working in this capacity. As such APP is used throughout this paper. In this study, the APPs were working in hospitals and any authorized activities were performed with indirect authorization using hospital-specific medical directives transferring authority to perform specific acts. APPs received training or were undergoing training in advanced arthritis care. They worked collaboratively with orthopaedic surgeons, most often working parallel to surgeons in the clinics. While there may be some institutional variation across hospitals in Ontario, the role described here is likely to be similar to an APP working in orthopaedic clinics elsewhere in the province.

Strengths of this study are the prospective research design and the pragmatic nature of the research embedded within a real-world pilot program. Data comparing APPs who had completed their training and those who had not fully completed training were analyzed and there were no differences in the results (data not reported). As noted above, the study also has limitations. A key limitation is the lack of a control group to compare outcomes to the traditional orthopaedic model of care. Participants may have seen saw other health professionals in the follow-up period and we are unable to attribute changes to the APP intervention. It is known that APPs recommended referral to community physiotherapy in less than one third of patients. However, data on participants’ baseline use of physiotherapy are not available. As a result, the uptake of recommendations is unclear and therefore, not included in the paper. Further study is required to understand patient outcomes in this model using APPs in comparison to other care delivery models in orthopaedics, and to determine to what extent the setting of the study in the context of an orthopaedic consultation in a tertiary care centre may have influenced the results. We are also relying on patient recall and are unable to link physiotherapist recommendations with patients’ recall of recommendations. In addition, the length of follow-up in the study was relatively short at six weeks and longer term follow-up is required to understand how the intervention may impact longer term outcomes. Finally, generalizability of this study is limited to English speaking adults with arthritis referred for potential TJR.

## Conclusion

In conclusion, new roles for health professionals have developed to address the increasing burden of chronic disease in the population in the face of health human resource challenges. This pilot study using a pre-post-intervention design showed promising findings suggesting that a relatively minimal intervention with an APP providing support for conservative management may be beneficial for some non-surgical patients with arthritis who were referred for consideration of surgery. Our study found that non-surgical patients had significant improvements in self-efficacy to manage their condition and time spent in exercise. Further research is required to examine long term patient outcomes in such an intervention using a randomized controlled trial design.

## Competing interests

The authors declare that they have no competing interests.

## Authors’ contributions

CM collaborated in conceptualizing the project, participated in its design and coordination, analyzed the data and drafted the manuscript. AD contributed to the conceptualization of the study, design, interpretation of data and helped to draft the manuscript. NM contributed to the conceptualization of the project, design, interpretation of data and reviewed the manuscript for intellectual content. EB contributed to the conception of the study, design, interpretation of data and assisted in drafting the manuscript. All authors read and approved the final manuscript.
